# Access to and engagement with healthcare services among women with Children’s Social Care involvement during the perinatal period who subsequently died: a confidential enquiry

**DOI:** 10.1136/bmjph-2025-003171

**Published:** 2026-04-10

**Authors:** Kaat De Backer, Emma Rose, Caroline Bull, Oluwaseun Labisi, Allison Marjorie Felker, Kirsty Kitchen, Claire Mason, Elsa Montgomery, Jane Sandall, Abigail Easter, Marian Knight, Nicola Vousden

**Affiliations:** 1 Department of Women and Children’s Health, King’s College London Faculty of Life Sciences & Medicine, London, England, UK; 2 Princess Alexandra Hospital NHS Trust, Harlow, England, UK; 3 Rosie Hospital, Cambridge University Hospitals NHS Foundation Trust, Cambridge, England, UK; 4 Croydon University Hospital, London, England, UK; 5 National Perinatal Epidemiology Unit, University of Oxford, Oxford, England, UK; 6 Birth Companions, London, UK; 7 Centre for Child and Family Justice Research, Lancaster University, Lancaster, UK; 8 Florence Nightingale Faculty of Nursing, Midwifery & Palliative Care, King’s College London, London, UK

**Keywords:** Health Services Accessibility, Public Health, Female, Sociodemographic Factors

## Abstract

**Introduction:**

In the last decade, UK maternal death surveillance data have shown that among the women who died during pregnancy and the year after birth (the perinatal period), the proportion of women with Children’s Social Care (CSC) involvement nearly doubled. Parental non-engagement in the context of CSC involvement has been described as a particular professional concern.

**Objective:**

To explore organisational and system barriers when accessing and engaging with healthcare services experienced by women with CSC involvement who subsequently died during the perinatal period.

**Methods:**

MBRRACE-UK national surveillance data were used to identify women who died during or in the year after pregnancy in the UK between 2014 and 2021 and who had CSC involvement. A confidential enquiry of healthcare records of a random sample of women with CSC involvement during pregnancy or in the year after the end of pregnancy and who subsequently died (n=47) was undertaken to explore barriers to care.

**Results:**

We identified four themes to describe the barriers faced by women with CSC involvement when accessing and engaging with healthcare services in the perinatal period: (1) burden of care, (2) disruption of care, (3) follow-up of non-attendance and (4) bias in care. Our findings highlighted the additional challenges that women had to contend with, while already facing multiple adversities in their daily lives.

**Conclusion:**

Our confidential enquiry found that the existing narrative of non-engagement among women with CSC involvement is unfounded for most women. Care for women with CSC involvement needs to be made trauma-informed, accessible and minimally disruptive.

**WHAT IS ALREADY KNOWN ABOUT THIS TOPIC**

- Previous UK confidential enquiries into maternal deaths have described how women with multiple adversity encounter many biases, affecting the quality of maternity care that they receive.
- A proportion of these women will also have Children’s Social Care (CSC) involvement, known to be associated with increased maternal mortality and morbidity and, for some, removal of their infant from their care.
- Little is known about the experiences of healthcare, in particular access and engagement with perinatal healthcare services for this group of marginalised mothers.

**WHAT THIS STUDY ADDS**

- Confidential enquiry methodology provides an opportunity to identify areas for care improvement for specific groups of maternal deaths and therefore has the potential to save lives. This study used confidential enquiry methodology to retrospectively review healthcare records of a random sample of 47 women with CSC involvement who subsequently died. We found that women with CSC involvement faced cumulative social adversity and a range of system barriers that hindered access and engagement. Care pathways were burdensome, fragmented and uncoordinated and compounded the adversities that women were already facing. A pervasive narrative of poor engagement with health services was reflected in women’s records, even when non-attendance was limited and not representative of women’s overall efforts to access and engage with services.

**HOW THIS STUDY MIGHT AFFECT RESEARCH, PRACTICE OR POLICY**

- Access to and engagement with healthcare services should be considered a reciprocal endeavour, and not solely attributed to parental efforts. A critical review of current maternity care pathways is urgently required, focusing on integration and coordination of care, to tailor care to the needs of women facing complex social adversity, reduce the burden of care and address existing health disparities that disproportionately affect this group of women.

## Introduction

 In recent months, the *British Medical Journal* published two calls for action to reduce maternal health inequalities for marginalised and underserved women.^1 2^ Among these, women with Children’s Social Care (CSC) involvement have long been neglected in research, clinical guidance and policy decisions. The 2025 UK national surveillance on maternal deaths (Mothers and Babies: Reducing Risk through Audits and Confidential Enquiries across the UK (MBRRACE-UK)) documented that 23% of women who died during or up to 6 weeks after pregnancy were known to CSC.^3^ Under UK children’s safeguarding legislation, CSC involvement can range from voluntary offers of support to mandatory child protection plans if a child is deemed to be at risk of significant harm. In the most serious circumstances, children are removed from their parents’ care through the family courts, which for infants can occur within hours or days after birth. The latest 2024 figures from the UK Office of National Statistics showed that more than 7000 unborn babies and almost 16 000 infants under the age of 1 year were considered to be at significant risk of harm by CSC and required some level of intervention.^4^

CSC involvement is known to complicate the ongoing relationship between parents and healthcare and social care professionals.^5^ Antenatal care attendance and engagement can be hindered due to fear of child removal.^6^ Distrust of healthcare professionals, previous negative experiences with CSC and internalised feelings of shame and undeserving motherhood have been reported as personal barriers to engagement with healthcare services during the perinatal period.^5^ Parental non-engagement with services due to fear of custody loss has been raised as a pressing issue and is often viewed by professionals as a resistance to help from services.^7 8^ Complex post-traumatic stress disorder as a result of chronic childhood maltreatment and its enduring impact on interpersonal relationships can make engagement with professionals challenging and anxiety-provoking.^9^ While some theoretical frameworks around parental engagement have focused on the reciprocal nature of engagement and the shared responsibility between parents and services to this effect,^5 10^ engagement has been most commonly attributed solely to the account of parents.^9^

A referral to CSC during pregnancy can set in motion a sudden influx of professionals that can lead women to feel overwhelmed.^11^ In contrast, once a child has been taken into care, mothers are often left unsupported and isolated, having to cope with the trauma of infant removal on their own.^9^ Qualitative studies from the UK, Australia and the USA have described feelings of shame, despair and loss of purpose among birth mothers who had a child removed.^12^,^15^ For some, the acute psychosocial crisis after infant removal might (re)trigger harmful coping strategies, such as drug and alcohol use.^12^ Quantitative studies using linked administrative data have associated infant removal with increased maternal morbidity and mortality post removal.^16^,^19^ For example, linked administrative data from Manitoba, Canada, where rates of children in care are among the highest in the world, identified 3.23-fold higher mortality rates of mothers whose child was taken into care when compared with a cohort of biological sisters whose child was not taken into care. Hazard Ratios (HR) were greater for avoidable causes of mortality than for unavoidable causes (adjusted HR 3.46, 95% CI 1.41 to 8.48 vs 2.92, 95% CI 1.01 to 8.44, respectively).^17^ Similar evidence for both mothers and fathers was found in a Swedish national cohort study, and highlighted increased risk of suicide among mothers, as well as ischaemic heart disease.^18^ In England, Pearson *et al* (2021) found a 2.15 times greater HR (95% CI 168 to 2.74) of dying in a South London cohort of women known to secondary mental health services who had their child removed.^19^

Also in the UK, yearly MBRRACE-UK reports have highlighted the high proportion of women who experienced separation from their infant due to safeguarding concerns among the women who died by suicide or substance use.^20^,^22^ The most recent MBRRACE-UK report also highlighted the challenges women with multiple disadvantages experience when accessing care.^22^ However, there has been no specific confidential enquiry from MBRRACE-UK focused on women with CSC involvement. Also, while CSC involvement is considered among women who died from certain causes, such as suicide or substance use, it is not typically considered in other instances of maternal death.

Confidential enquiry methods seek to identify common patterns in mortality cases, in order to identify opportunities for care improvement and not to apportion individual blame. As such, they have proven to be a powerful tool to foster change and reduce mortality and morbidity, including in the UK through yearly MBRRACE-UK Confidential Enquiries into maternal deaths, as well as in the USA through the Fetal Infant Mortality Review system.^23^ However, confidential enquiries have not yet explored the quality of care received by women with CSC involvement in detail. To address this evidence gap, we conducted the first retrospective cohort study of UK maternal mortality surveillance data and a confidential enquiry into the care of women with CSC involvement. A first paper, investigating the characteristics, outcomes and experiences of care of women with CSC involvement who subsequently died during or in the year after the end of pregnancy, has been published elsewhere.^24^ The aim of this second paper was to explore the organisational and system barriers that women experienced when accessing and engaging with perinatal healthcare services prior to their death.

## Materials and methods

### Data source and cohort identification

MBRRACE-UK is the national consortium responsible for delivering the Maternal, Newborn and Infant Clinical Outcome Review Programme. MBRRACE-UK reviews all maternal deaths in the UK during or up to 1 year after pregnancy,^25^ in accordance with WHO Maternal Death Surveillance and Response (MDSR) guidance.^26^ MBRRACE-UK is notified of maternal deaths through a variety of processes and deaths are cross-checked with national vital statistics records to ensure completeness. Full medical records, including maternity notes and records from various healthcare providers, such as women’s general practitioner (GP), other hospitals, mental health or social care services, are anonymised and uploaded to a secure online portal. The notifying practitioner is also required to complete the MBRRACE-UK surveillance form, containing a range of questions on demographic and clinical characteristics, pregnancy and birth outcomes, CSC involvement and cause of death.

For this study, two questions from the MBRRACE-UK surveillance form were used to identify the cohort of women with CSC involvement who died during or within a year of their pregnancy in the period 2014–2021: (1) ‘*Was the woman known to CSC?*’ (2) ‘*Was the newborn infant taken or to be taken into care?*’ We only included case notes of women for whom this information was completed on the surveillance form; case notes of women for whom this information was missing were excluded. We defined CSC involvement as any active or ongoing involvement of CSC, during pregnancy and/or the postnatal period for the unborn or newborn child, prior to the woman’s death. This included various levels of CSC involvement, as stipulated by the corresponding sections of the Children’s Act 1989, such as voluntary offers of support (s.17), mandatory child protection (s.47), court ordered removals (s.31) or voluntary arrangements between CSC and the child’s parent(s) (s.20).^27^

To ensure the confidential enquiry reviewed a breadth of CSC involvement, four subgroups were created based on level of involvement (ie, whether the infant went into care or not, by court order or parental agreement) and time of death (early or late maternal death). For late maternal deaths, sampling was also based on specific causes of death (ie, deaths by suicide, substance use and homicide). This decision was made in consultation with the wider steering group and based on the evidence from previous MBRRACE-UK reports regarding over-representation of women with CSC involvement among these lead causes of late maternal deaths.^28^ For each of the four subgroups, 20 case notes were randomly sampled for an in-depth case note review using a random number generator. After sampling, the demographics of sampled cases were reviewed to ensure that they were representative of the whole subgroup regarding year of death (2014–2021), as well as geographical representation across England, Scotland and Wales, in keeping with known maternal mortality rates. Where initial random sampling resulted in skewed, non-representative cases, random sampling was re-done.

The terms ‘pregnant women’ and ‘mothers’ will be used throughout this paper as there was no evidence from medical records to suggest that those included in the study identified as anything else. However, the authors recognise not everyone who is pregnant or giving birth will identify as a woman or a mother.

### Confidential enquiry methodology

The WHO defines a confidential enquiry into maternal deaths as ‘a systematic multidisciplinary anonymous investigation of all or a representative sample of maternal deaths occurring at an area, regional or national level which identifies the numbers, causes and avoidable or remediable factors associated with them’.^29^ Confidential enquiry methodology uses a combination of qualitative and quantitative analysis methods and takes account of the medical and non-medical factors that led to a woman’s death. Data on individual cases are aggregated and can show common factors for which remedial action may be possible. The strength of confidential enquiry methodology lies in its potential to save lives, by identification of areas for care improvement for specific groups of maternal deaths.^29^ For this study, our sampling focus was on women with CSC involvement and our priority was to identify the barriers these women experience when accessing and engaging with healthcare services during pregnancy or the year after birth, prior to their death. Through the MBRRACE-UK portal, various qualitative data sources were available, such as clinical notes and documentation by healthcare professionals during ‘live’ healthcare encounters, reflective accounts that healthcare professionals are invited to submit by MBRRACE-UK when they were involved in the care of a woman who died, postmortem and coroners’ reports and, where available, reports of child protection conferences.

In order to adopt a rigorous and systematic approach, we designed a data extraction template to facilitate extraction of information on demographic and clinical characteristics, complex social risk factors and CSC involvement, as well as key themes of what good care for women with social care involvement should look like. These themes were based on previous relevant guidance, such as the Birth Companions’ ‘Birth Charter for women with involvement from children’s social care’^30^ and the ‘Born into Care best practice guidelines for when the State intervenes at birth’.^31^ A list of prompts accompanied the template, to ensure a similar approach to data collection and extraction was taken (see online supplemental material S1). Relevant information per key theme was summarised in narrative form by each assessor onto the spreadsheet. All medical records were reviewed by one assessor, with two-thirds of records being reviewed by a second assessor. Assessors discussed each case collectively, identifying minimal differences in <5% of extracted fields and agreed on key findings by consensus. Findings were thematically grouped and themes were iteratively discussed with the wider supervisory team, a steering group as well as with a group of women with lived experience of infant removal, to allow sense-checking and prioritisation of key themes and subthemes.

In accordance with WHO MDSR guidance, the research team consisted of multidisciplinary expertise to assess medical and non-medical factors influencing care.^26^ This meant the research team consisted of researchers and clinicians from a range of professional backgrounds (obstetrics, midwifery, public health and social science) and with expertise in perinatal mental health, domestic abuse, substance use, safeguarding and quality standards.

### Lived experience involvement

This study was part of a doctoral study, funded by the National Institute for Health and Care Research (MUMS@RISC, NIHR302565). The MUMS@RISC study centres the voices of women with lived experience through regular consultation of the MUMS@RISC advisory panel, consisting of six women with lived experience of infant removal. The panel was involved in the design of the data extraction template, analysis and sense-checking of the findings. The latter was also supported by the Birth Companions’ Lived Experience Team and the HOPE Mothers group, a collective of birth mothers who had an infant removed from their care.

## Results

**Figure 1:**
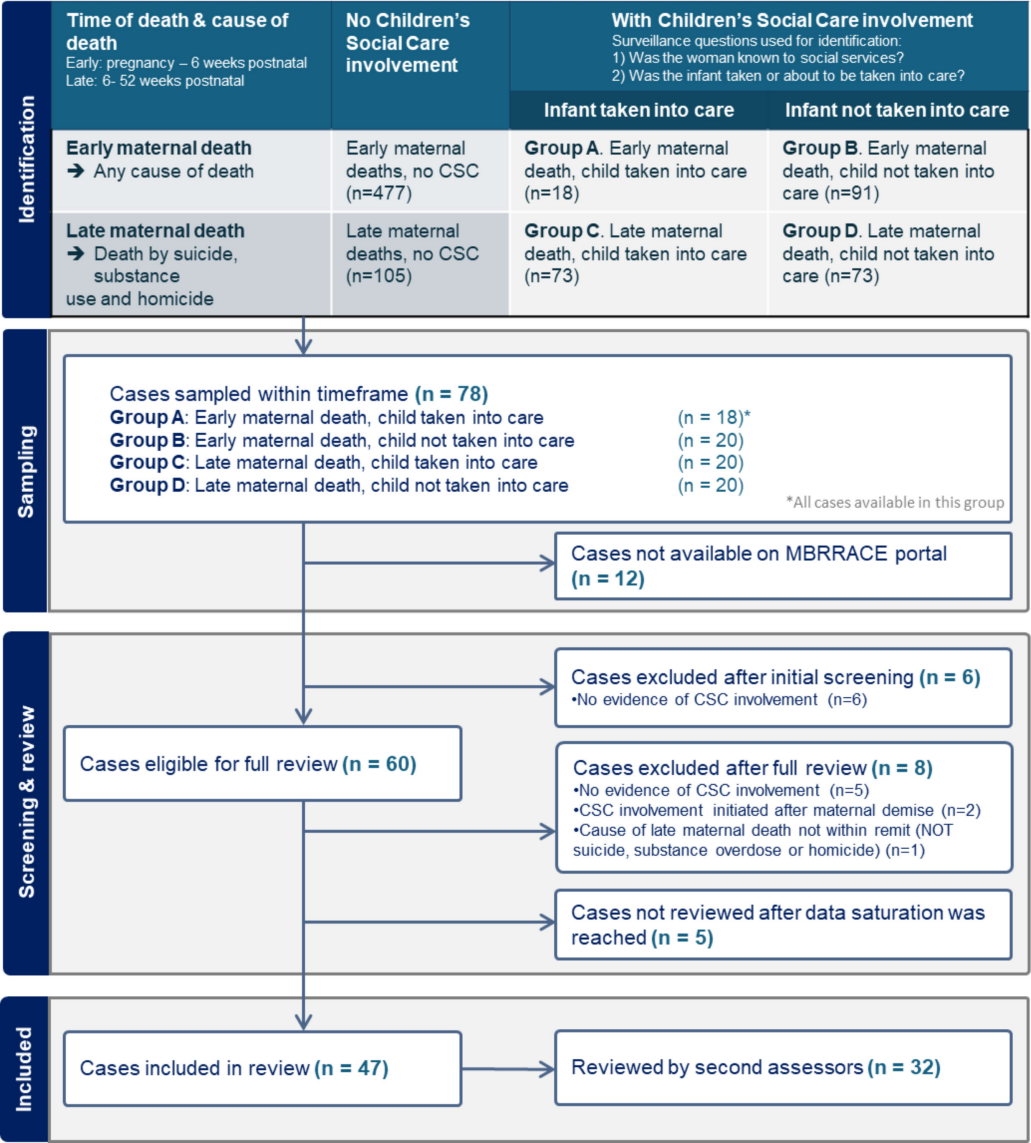
Sampling and inclusion for confidential enquiry. CSC, Children’s Social Care; MBRRACE, Mothers and Babies: Reducing Risk through Audits and Confidential Enquiries across the UK.

Of the 78 sampled cases, 12 cases were not available at the time of review. These were records of women who died either in pregnancy or in the postnatal period whose relevant records were not yet available to the MBRRACE-UK team. A further six cases were excluded as the information on the surveillance form regarding CSC involvement had been completed incorrectly. After initial screening of these cases, there was no evidence found of CSC involvement during the perinatal period; hence, a full in-depth case note review was not appropriate. A total of 55 cases were fully reviewed, of which eight were excluded after review. Records of 47 women who died during or in the year after pregnancy were included in the confidential enquiry, with figure 1 showing the sampling, screening and reviewing process. The decision to cease further review of the remaining sampled records was based on theme saturation, time required to complete the reviews, as well as to minimise vicarious trauma among the assessors’ team, in view of the cumulative exposure to the distressing content in care records.

### Maternal demographic characteristics

Data on maternal characteristics were obtained from routinely collected surveillance information and supplemented with additional data from maternal care notes and are presented in table 1.

**Table 1:** Maternal characteristics of the women included in the confidential enquiry

Demographic characteristics	n=47 (%)
Ethnicity	
White Asian/Asian British/Asian Welsh Black/Black British/Black Welsh/African Mixed or multiple ethnic groups Other ethnic group Missing	39 (83.0%) 3 (6.4%) 1 (2.1%) 1 (2.1%) 1 (2.1%) 2 (4.3%)
Age at time of death Mean_Age_ 30.4 years, range 16–41	
Under 20 years 20–24 years 25–34 years 35–39 years 40 years or older	3 (6.4%) 8 (17.0%) 23 (48.9%) 11 (23.4%) 2 (4.3%)
Parity at time of death	
P0 P1 P2 P3 P4 P5 or more	3 (6.4%) 11 (23.4%) 14 (29.8%) 7 (14.9%) 5 (10.7%) 7 (14.9%)
Time of death	
During pregnancy Early maternal death <6 weeks after end of pregnancy Late maternal death >6 weeks after end of pregancy	10 (21.3%) 13 (27.7%) 24 (51.1%)
Relationship status	
Single In relationship Married Separating during the perinatal period	7 (14.9%) 32 (68.1%) 4 (8.5%) 4 (8.5%)
Living arrangements	
Living alone Living with partner Living with parents or extended family Supported living accommodation Unstable housing situation In custody Missing	13 (27.7%) 16 (34.0%) 6 (12.8%) 2 (4.3%) 4 (8.5%) 1 (2.1%) 5 (10.6%)
Employment status	
Unemployed (including in custody) In education Permanently sick/disabled Housewife/looking after family No rights to work Missing	35 (74.5%) 1 (2.1%) 2 (4.3%) 4 (8.5%) 1 (2.1%) 4 (8.5%)
**Clinical characteristics**	
Late booking (>13 weeks gestational age)	16 (34.0%)
Antenatal care attendance*	
Regular Inconsistent Not applicable	35 (75%) 11 (23%) 1 (2%)
Known medical risk factors	35 (74.5%)
Known obstetric risk factors	39 (83.0%)
Known mental illness	39 (83.0%)
Causes of death	

	Early maternal deaths (pregnancy–6 weeks postnatal) n=23 (%)	Late maternal deaths (6 weeks–1 year postnatal)† n=24 (%)
Psychiatric – suicide	4 (17.4%)	9 (37.5%)
Psychiatric – substance overdose	2 (8.7%)	14 (58.3%)
Homicide	1 (4.4%)	1 (4.2%)
Cardiac disease	4 (17.4%)	–
COVID-19 related	1 (4.4%)	–
Thrombosis and thromboembolism	6 (26.1%)	–
Neurological	1 (4.4%)	–
Sepsis	2 (8.7%)	–
Unascertained	1 (4.4%)	–
Other	1 (4.4%)	–

* Attendance was viewed in line with the National Institute for Health and Care Excellence Clinical Guideline 201,^47^ which stipulates a schedule of antenatal appointments for nulliparous and multiparous women. Antenatal care was considered regular when women were seen at least once by a maternal care professional around the time of these recommended contacts. Attendance was considered regular even if booking had occurred later than the recommended 13 weeks, as long as this was before 20 weeks and was due to being unaware or ambivalent towards pregnancy. Attendance was considered ‘not applicable’ if the woman died before the recommended booking appointment could occur.

† Late maternal death cases were sampled by causes of death by suicide, substance overdose and homicide, due to their strong association with Children’s Social Care involvement in the late postnatal period.

Women were predominantly multiparous (33/47), of white ethnicity (39/47), with a mean age of 30.4 years (range 16–41 years). Most women were in a relationship (36/47) and almost half of those were co-habiting with their partner (16/36). Two-thirds of women were unemployed, and an additional three women were unable to work due to being in custody for some (n=2) or all (n=1) of their pregnancy.

About one-third of the women had a booking appointment later than 13 weeks gestation. The majority of women had a significant medical (35/47), obstetric (39/47) or mental health (39/47) history, requiring a consultant-led care pathway for their pregnancy care.

For women who died during or within 6 weeks after the end of pregnancy (n=23), thrombosis and thromboembolism was the leading cause of death (6/23), with suicide and cardiac disease being the second most common causes of death (4/23). Substance overdose and sepsis (2/23) were the subsequent most common causes of death for this randomly sampled group. In the late pregnancy-associated death cohort (n=24), which had been specifically sampled for psychiatric causes and homicide, almost two thirds of the women died due to substance use (14/24), with nine women dying by suicide. In both the early maternal death and late maternal death sub-groups, one woman died by homicide.

The cumulative burden of complex social risk factors can be found in table 2. Almost half of the women faced five or more complex social risk factors. In two-thirds of case notes, evidence of ongoing domestic abuse was found, with similar trends for perinatal substance misuse, homelessness and housing issues and significant childhood adversity. Referrals to CSC occurred for most women during the first trimester and were mostly completed for the unborn or the infant only. In almost half of instances, the infant was taken into the care of the local authority. Where women had previous children, almost two-thirds of these mothers did not have their children in their care.

**Table 2:** Social complexity and Children’s Social Care (CSC) involvement

Social risk factors	Total cohort n=47 (%)
Previous social issues	
Previous mental health issues Previous domestic abuse Previous substance misuse Previous criminal justice involvement	38 (80.9%) 31 (66.0%) 28 (59.6%) 19 (40.3%)
Ongoing complex social risk factors	
Perinatal mental health issues Homelessness or insecure housing Issues with partner or father of infant* Perinatal domestic abuse Significant adverse childhood experiences Perinatal substance misuse Significant financial need Absence of social support Learning difficulties (including unable to read or write) Perinatal criminal justice involvement Care experienced Physical disability Young (20 years or younger) Unable to speak or understand English Recent migrant (<1 year)	34 (72.3%) 30 (63.8%) 30 (63.8%) 28 (59.6%) 28 (59.6%) 27 (57.5%) 22 (46.8%) 17 (36.2%) 15 (31.9%) 11 (23.4%) 10 (21.3%) 4 (8.5%) 4 (8.5%) 2 (4.3%) 1 (2.1%)
Social complexity and CSC involvement	
Complex social risk factors†	
Fewer than three risk factors Three to five risk factors More than five risk factors	8 (17.0%) 18 (38.3%) 21 (44.7%)
Referred in first trimester	33 (70.2%)
Referred to social services for:	
Mother Unborn or infant Both Older children	1 (2.1%) 39 (82.9%) 6 (12.8%) 1 (2.1%)
Infant taken into care	21 (44.7%)
Previous child(ren) not in parental care (if multiparous, n=36)	24 (66.7%)

* Includes mental health issues, contact with criminal justice system and substance misuse.

† Risk factors included: perinatal domestic abuse, perinatal mental health issues, perinatal substance misuse, criminal justice system (CJS) involvement during the perinatal period, homeless or insecure housing, young age (20 years or younger at time of death), learning difficulties, physical disabilities, absence of social support, significant financial need, recent migrant (less than 1 year), unable to speak or understand English, care-leaver, significant adverse childhood experiences).

### Barriers to care for women with CSC involvement

In the following paragraphs, we provide an overview of the organisational and system barriers faced by women with CSC involvement when accessing and engaging with healthcare services. To illustrate these themes, anonymised vignettes of cases included in this review, as well as visual displays of care pathways, are provided in conjunction with relevant paragraphs.

#### Burden of care

Most women in the cohort presented with complex social adversity and medical or obstetric comorbidity (table 2). These risk factors often triggered a range of health and CSC referrals and a sudden influx of professionals from various services and agencies during pregnancy. As a result, women were given a high number of antenatal appointments across different services, placing significant burden on women to attend such an intense antenatal appointment schedule. There was evidence from the review that appointments were often uncoordinated and with limited professionals’ awareness of other services being involved in women’s care. Lack of coordination resulted at times in multiple appointments being scheduled in different locations on the same day, or in close succession across subsequent days. The case note review found evidence that most women consistently attended these appointments, which for several women exceeded over 30 different contacts during a pregnancy episode. Vignette 1 illustrates the burdensome antenatal appointment schedule that many women were faced with.

#### Vignette 1

A woman attended for her maternity care during the first trimester. She was already known to CSC for her older child. She had a history of alcohol misuse, resulting in liver disease and developed gestational diabetes during pregnancy. CSC extended their involvement to include her unborn child on a Child Protection plan. She had over 40 antenatal contacts with maternity healthcare professionals. A clear obstetric or safeguarding plan could not be identified in the medical notes. Her maternity record indicated she was also under the care of a specialist addiction service and supported by a mental healthcare coordinator, with whom she was expected to have weekly contact. On three occasions, miscommunication by the hospital was evident, leading to the woman missing appointments. ‘Failed to attend’ was recorded in her maternity notes.

**Figure 2:**
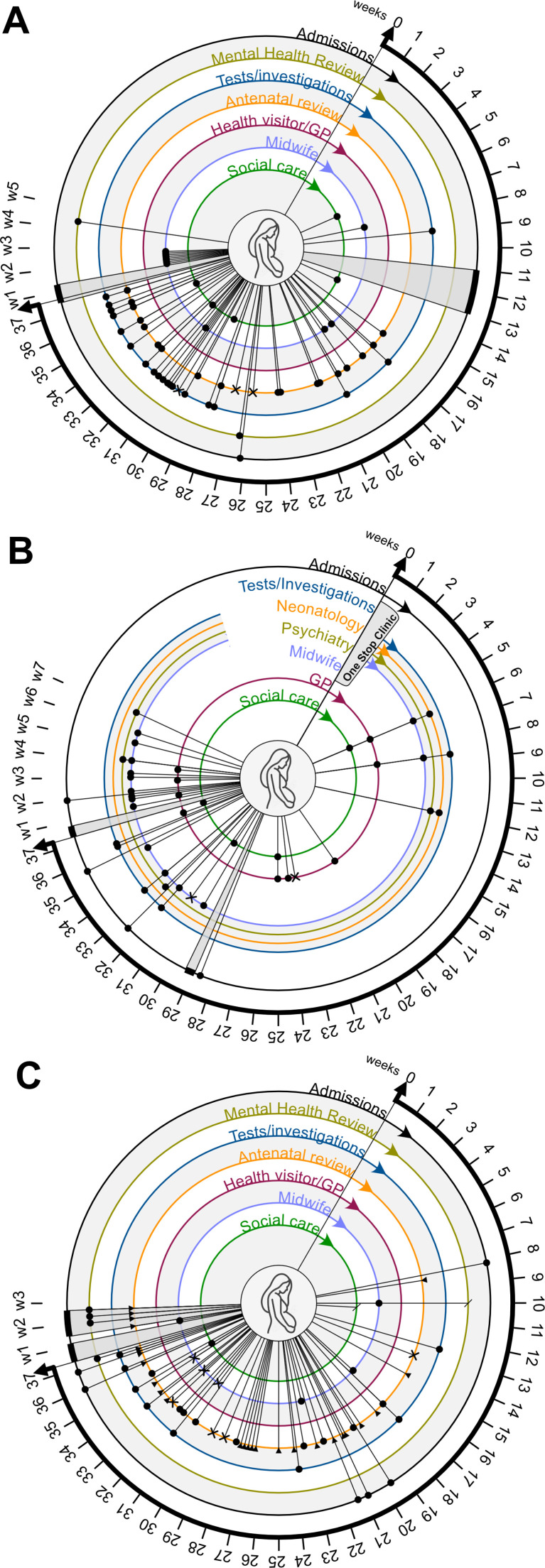
Visual display of care pathways. Visual display of care during pregnancy and the first postnatal month, at different timepoints (weeks of gestation), illustrating burden of care (A), a one-stop-shop approach (B) and non-attendance and subsequent labelling of poor attendance (C). Appointments with non-attendance are marked with **x**. Sections in grey represent inpatient admissions. Telephone contacts are marked with 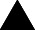. Referrals with no further action are marked with /. GP, general practitioner.

When reviewing the case notes, the high frequency of reviews from the diabetes and obstetric team, with multiple ultrasounds and additional cardiotocographs, appeared uncoordinated and reactive, with no regard for the financial and social barriers that the woman had to overcome to travel to hospital with such frequency. Assessors found no evidence that maternity services were aware of the ongoing involvement of addiction services and triggers for relapse in drinking were not clearly identified or included in any obstetric or safeguarding plans. A visual timeline of the woman’s contact with services during pregnancy and the first postnatal month can be found in figure 2A.

Assessors felt that a coordinated, multidisciplinary approach might have reduced the burden of uncoordinated appointments and could have facilitated information-sharing among professionals to allow for appropriate, individualised pregnancy and birth planning with risk mitigation for alcohol dependency relapse.

Three women included in the case note review were offered a so-called ‘one-stop shop’ approach, with input from different professionals at one given time and location. One example of this approach is available in Vignette 2. This woman’s antenatal care took place in a ‘One Stop Clinic’, bringing a strong multidisciplinary focus to her care, with continuity of midwifery care by a designated specialist midwife across the perinatal period. She was referred to multiple additional services, such as a local perinatal mental health team, and important information was available to all involved in her care.

#### Vignette 2

A pregnant woman presented with longstanding alcohol and drug use, mental health problems and homelessness. Her previous children had been removed at birth in view of these issues. She was uncertain about continuing her pregnancy, which delayed commencement of a designated care pathway for women with substance misuse or homelessness. Her antenatal care was coordinated by a specialist midwife who was part of a multidisciplinary antenatal team, which included a range of specialists, including, drug and alcohol services. There was additional input from a local charity for homeless people. Her baby was taken into foster care shortly after birth. Her specialist midwife continued to see her for 6 weeks after the birth. Despite this multidisciplinary care, she died 2 months after birth from a substance overdose.

Throughout the woman’s pregnancy and postnatal care, the case note review found clear evidence of regular multidisciplinary meetings, for instance through email exchanges between health and social care professionals across primary and secondary care. The coordinated approach enabled access to specialist input as soon as the need arose. Figure 2B provides a visual display of her care journey. Although antenatal records were partially missing for care in the second trimester, the available information on the overall care journey demonstrated flexibility and synchronisation of care, where antenatal contacts became opportunities for review by a range of professionals at one given time.

#### Disruption of care

While the one-stop-shop approach described above meant women were receiving care from the same midwife and multidisciplinary team throughout their pregnancy, other women faced additional challenges due to high turnover of professionals or teams involved. Some women were discharged during pregnancy from mental health teams that had been involved pre-pregnancy, as their mental health or addiction treatment had come to an end, and they were considered ‘stable’ or ‘in recovery’. The consistent care they had received prior to pregnancy suddenly ceased, and with that, established relationships and trust from an allocated care coordinator, as well as crucial insight into recognition of first warning symptoms or deteriorating mental illness was lost. For other women, disruption was caused due to relocation, incarceration, refuge placement, temporary housing or inpatient care in psychiatric or acute hospital facilities. Others were discharged from services once their infant was no longer in their care, with services abruptly ending their involvement with the mother. At each point of transition, care was at risk of disruption, especially when handovers between different care providers were incomplete or delayed, and care fragmentation was exacerbated.

#### Follow-up of non-attendance

A third theme we identified was the ineffective follow-up of non-attendance. Once women had initiated antenatal care, the majority attended most or all antenatal appointments. However, as described above, for some women the expectations of a fragmented and uncoordinated appointment schedule were unattainable, leading to non-attendance. While there were some good examples of assertive outreach and flexibility to support further access and engagement, we also found areas where processes could be improved to check in with women and offer alternative arrangements that were manageable to attend. In some records, there was evidence that women were discharged from specialist outpatient clinics and referred back to their GP after a single non-attendance, rather than being offered a new appointment. This meant crucial expert input became even more inaccessible and was for some women no longer pursued. Some women were even discharged from their GP surgery due to persistent non-attendance, creating greater disruption and obstacles. Opportunities to explore why women had not attended and how these barriers might be overcome were missed. Medical records contained limited clinical documentation to evidence a professional understanding of women’s complex social circumstances, previous adverse life events and trauma and its profound, enduring impact on women’s ability to access and engage with healthcare.

For several women, non-attendance was met with a punitive stance through escalation to the social worker involved. This approach disregarded the overall levels of engagement that women had displayed throughout their pregnancy care. As a result, many women were labelled ‘late booker’ or ‘poor attender’ in clinical documentation. All too often, handovers with reductive descriptions, such as ‘poor engagement’, ‘late booker’ and ‘poor historian’, were repeatedly copied in subsequent clinical handover notes, even when women had attended multiple appointments since. The review found minimal evidence of healthcare professionals’ consideration regarding the financial and logistical implications of multiple and frequent hospital-based appointments, in particular, among women with caring responsibilities for older children when social support around them was limited.

#### Vignette 3

A woman was considered to have a medically high-risk pregnancy at her initial contact with maternity services. Thromboprophylaxis was prescribed and a referral to CSC was made, with no further action. The woman attended almost all of her hospital-based appointments, however, her engagement with community midwifery was inconsistent and clinical documentation described her as a ‘poor attender in the community’. Community midwifery staff did not explore the reasons for non-attendance until late in the third trimester, when the woman disclosed restricted mobility had prevented her from attending the local clinic. Similarly, there was no evidence of any conversations around the challenges she faced at home. She remained unsupported until she self-referred to family support services in the third trimester. The family, including the unborn baby, were subsequently escalated to social services by the early help worker, only a few weeks prior to birth.

In contrast to Vignette 1 where multiple services were involved in antenatal care, the woman in Vignette 3 only had maternity services involved during pregnancy, yet her antenatal care was similarly fragmented and uncoordinated. Figure 2C gives an overview of the antenatal care schedule. Earlier referrals to mental health support and CSC were not actioned, leading to rushed involvement in the weeks around birth.

#### Bias in care

This review found evidence of professional bias and stigma towards women with CSC involvement, which are shown in table 3. These examples were taken from repeated documentation across the sample. In almost 75% of case notes (35/47), one or more of the following types of bias were found.

**Table 3:** Evidence of professional bias and stigma across a majority of women’s case notes

Type of bias or stigma*	Examples from case notes
Use of generalisations or platitudes, stripping women of their individuality	*‘Drug user’, ‘addict’, ‘learning difficulties person’, ‘non attender’, ‘poor historian’*
Unnecessary use of quotation marks, that could be interpreted as being critical to women’s accounts	*‘Woman claims to have “lost” her medication’*
Use of exclamation marks to sensationalise information	*‘Hiding out in the area!’*
Referencing historic behaviour when there is no clinical indication to do so and presenting this as an ongoing risk	*‘Ex drug-abuser’, ‘ex sex worker’*
Responsibility for the harm from domestic abuse entirely or partially attributed to the woman while the perpetrator’s abusive behaviour is minimised or absent	*‘Inability to maintain personal boundaries’, ‘chaotic interpersonal relationships’*
Absence of trauma-informed approach to understand previous trauma and adverse life events	*‘Chaotic lifestyle’, ‘dysfunctional’*
Professional complacency to provide optimal support	*‘There would appear to be some inevitability about this maternal death’ (from clinician’s reflective account)*

* Evidence of judgement and bias in clinical records was summarised in narrative form, alongside examples found in clinical documentation. Types of biases were thematically grouped, and iteratively discussed during assessors’ meetings, with the wider supervisory team, the steering group, as well as with women with lived experience.

Professional bias towards women with CSC involvement was most frequently observed by the use of dehumanising and stigmatising language (15/47). This was most evident in the care of women with substance dependency and those who experienced domestic abuse. Our confidential enquiry found limited evidence of trauma-informed approaches, exploration of and insight into women’s complex social circumstances and previous adverse life events. It is important to note that current guidelines to support trauma-informed approaches in healthcare settings only came into effect after the timeframe of the study cohort (2014–2021).

## Discussion

This confidential enquiry found some evidence of high quality, personalised care where professionals provided assertive outreach and worked with women to tailor healthcare around their individual circumstances and to facilitate uptake of and continued engagement with antenatal care. However, many women whose care was reviewed faced burdens to accessing care through numerous and uncoordinated appointments and disrupted care. There was also evidence of ineffective processes to follow-up non-attendance and professional stigma and bias towards women with CSC involvement evident in clinicians’ notes. Overall, there seemed to be limited professional awareness of the enduring impact of social adversity on maternal outcomes,^32 33^ women’s mental health,^34^ maladaptive coping strategies^35^ and availability to engage with perinatal healthcare.^36^

While some women in the cohort initiated their antenatal care in the later stages of pregnancy, two-thirds of women attended their initial antenatal appointment before 13 weeks gestation as recommended.^37^ For those that delayed antenatal care initiation beyond this point, uncertainty around whether or not to continue with the pregnancy was a common feature. The reductive label of ‘late booker’ did not account for the dilemma that many women were facing and instead created a narrative of poor engagement from the very onset of contact with maternity services. This narrative of poor engagement was amplified when women missed subsequent antenatal appointments. None of the women in this confidential enquiry had a total absence of antenatal care. Two women required assertive outreach to complete their booking appointment, with minimal antenatal checks attended during the remainder of their pregnancy. The majority of women attended antenatal appointment schedules that by far exceeded routine care pathways for nulliparous or multiparous women with medically low risk pregnancies.^38^ The complexity and burden of appointments was often not appropriately acknowledged or addressed by professionals involved. Even in these circumstances, a single non-attendance often resulted in clinical documentation reflecting a pervasive narrative of poor engagement of women with CSC involvement. As such, women with complex social adversity were expected to meet higher expectations when it comes to non-attendance than women who do not have the same barriers to accessing and engaging with care.

A growing body of literature has looked at parental non-engagement during mandatory child protection processes with a focus on interactions between parents and social workers. This has brought nuance to the discourse and has contextualised parental non-engagement through a lens of previous trauma and adversity,^9^ attachment theory,^39^ historic exploitation and colonialism,^40^ and professional misconception.^41^ While this has fostered an increased understanding of the enduring impact of trauma on trust-building with professionals in social work practice,^9 10 41 42^ a similar shift in narrative has been slow to observe in maternity services. It is therefore not surprising that our care review found evidence across the included timeframe of stigmatising terminology, professional bias and limited adoption of trauma-informed approaches. Calls for embedding trauma-informed approaches in maternity care have been increasing in the last few years, both by researchers,^43^ third sector organisations^30^ and through best practice guidance.^31 44^ Moving away from a culture of stereotyping and dehumanising women with CSC involvement towards a genuine professional understanding of complex and repeated trauma is urgently required to improve care for this group of women.

In light of the themes identified in this review, engagement with services for this group of mothers would benefit from application of the theoretical framework of ‘Burden of Treatment’.^45^ A growing body of literature has looked into the disruptive effects of adherence to medical treatment on people’s working and social lives and the burden this puts on patients.^45 46^ Increasing demands placed by healthcare services on patients with chronic conditions in terms of attendance, hidden costs of getting to clinic appointments and taking time off work, digital literacy and treatment adherence (for instance, by self-administering thromboprophylaxis), in fact, induce non-adherence and non-engagement.^45^ This too is relevant for pregnant women with medical, obstetric or psychiatric comorbidity, who face increased medical surveillance during pregnancy, as per the majority of this review’s cohort. Ongoing CSC involvement, with an additional schedule of social worker home visits, regular core groups and child protection conferences, exacerbates the ‘Burden of Treatment’. Cognitive and logistic availability to meet this burden is for this group of women further impaired by complex social adversity, such as insecure housing, deprivation and domestic abuse. With this in mind, our review found that one-stop-shop approaches may provide an opportunity to reduce the burden placed on women and offer access to a range of professionals at a single point of contact. Further research is required to formally evaluate the efficacy of these care models and how to improve and integrate perinatal multidisciplinary care to ease the burden of both medical and social care interventions on women who already have so many other challenges to contend with.

### Strengths and limitations

Our study is the first UK-based confidential enquiry into the care of women with CSC involvement who died during or within the year after the end of pregnancy. It builds on previous work of the MBRRACE-UK collaboration, yet brings extended information and insights into the specific complexities and adversities that this group of women experiences when accessing and engaging with healthcare. The confidential enquiry sample equated to approximately 18% of the total number of women with CSC involvement who died during the 2014–2021 timeframe in each of the sub-groups. As such, it provides insights into the care demands experienced by this group of women, and the barriers they faced to access and engage with services. However, it is important to acknowledge that confidential enquiry methods are limited by what is available in clinical records, documented by healthcare professionals working in a system under strain. There might have been additional important nuances of care, such as compassion, non-verbal communication and biased attitudes that were undocumented and therefore missing for this review. In addition, at times, clinical documentation was done in illegible handwriting, or records had missing pages or high levels of redacted information. While this complicated interpretation and data extraction in a small number of case notes, it did not affect the overall findings. Missing data on social risk factors was due to inconsistent documentation in maternity notes. This was also relevant to missing mental health records or social care records. Full access to these records would have provided a more accurate picture of the true burden of care on women with CSC involvement. Lived experience involvement was integrated into the design, analysis and sense-checking of the findings and our methods for documentary analysis of records were rigorous and robust. This was confirmed during team discussions, with minimal discrepancies identified in less than 5% of data extraction fields. Assessors’ well-being was carefully considered, with access to peer support and supervision. Our methods for this confidential enquiry were robust, building on the extensive experience in the team with this type of research method. It should also be recognised that the findings will be grounded in the multidisciplinary domains of experiences of the assessors who contributed.

## Conclusion

This study is the first of its kind in the UK to retrospectively review the care of women with CSC involvement who subsequently died. It significantly enhances our understanding of the complex care journeys that women navigate during pregnancy and the postnatal period. This study shows how access and engagement is hindered by the cumulative burden of social risk factors, uncoordinated and disrupted care and professional bias. A pervasive narrative of poor engagement was reflected in women’s records, even when their non-attendance was limited and not representative of women’s overall efforts to access and engage with healthcare. Trauma-informed approaches to understand how previous trauma and ongoing adversities can impact engagement are urgently required to enforce a paradigm shift among healthcare professionals towards this group of women. Best practice models that reduce the burden of care, through integrated care models, should be further tested and adapted to tailor multidisciplinary care around the complex physical, mental and social needs of this vulnerable group of women.

## Supplementary material

10.1136/bmjph-2025-003171 online supplemental file 1

## Data Availability

Data are available upon reasonable request.
