# Supplementary material for: Access to and engagement with healthcare services among women with Children’s Social Care involvement during the perinatal period who subsequently died: a confidential enquiry
Source: BMJ Public Health. 2026 Apr 10;4(2):e003171. doi: 10.1136/bmjph-2025-003171 (PMC13084787; doi:10.1136/bmjph-2025-003171)
Supplement: online supplemental file 1 [file bmjph-4-2-s001.docx]

*Supplementary Table S1 Data extraction Template*

| **Case ID** | | | |  | |  |
| --- | --- | --- | --- | --- | --- | --- |
| **FROM DATA SURVEILLACE FORM** | **Cause and place of death** | | |  | |  |
|  | **Early or late maternal death** | | Pregnancy (gestation): |  | |  |
|  |  |  | Postnatal (w/m PN): |  | |  |
|  | **Summary of case and care**  (only to be used as prompts to identify possible vignettes and demonstrate key findings) | | |  | |  |
|  | **Demographics** | Age at death | |  | |  |
|  |  | Ethnicity | |  | |  |
|  |  | Country of birth | |  | |  |
|  |  | Socio-economic status (IMD quintile) | |  | |  |
|  |  | Employment status | |  | |  |
|  |  | Partner status e.g. single or has a partner/spouse | |  | |  |
|  |  | BMI at booking | |  | |  |
|  |  | Gestational age at booking | |  | |  |
|  | **Medical/obstetric risk factors** | | | **Yes/No** | **Detail** |  |
|  | **Medical**  **history** | Gravidity & Parity | |  |  |  |
|  |  | History of stillbirth/Neonatal death/SIDS | |  |  |  |
|  |  | Known medical conditions | |  |  |  |
|  |  | Known obstetric risk factors | |  |  |  |
|  |  | Known mental illness | |  |  |  |
| **Complex social factors of multiple disadvantage*** | | | | **Present (Y/N/NA)**** | **Actions taken/support offered/support in place** |  |
| **Complex social risk factors** | Domestic abuse  (If possible provide information about perpetrator and type of abuse) | | |  |  |  |
|  | Substance misuse  (please specify: alcohol/illicit drugs/over the counter or prescription drugs used in harmful way) | | |  |  |  |
|  | Mental Health issues | | |  |  |  |
|  | Criminal justice involvement | | |  |  |  |
|  | Homelessness/Insecure housing | | |  |  |  |
|  | Young (<20yrs) | | |  |  |  |
|  | Learning difficulties or disability | | |  |  |  |
|  | Physical disabilities | | |  |  |  |
|  | Absence of social support (partner/family) | | |  |  |  |
|  | Significant financial need | | |  |  |  |
|  | Recent migrant (<1yr) | | |  |  |  |
|  | Unable to speak or understand English | | |  |  |  |
|  | Care experienced (as child/adolescent) | | |  |  |  |
|  | Concerns re partner (such as paternal substance misuse/MH/CJS) | | |  |  |  |
|  | Other | | |  |  |  |
| **Summary of social services involvement**  (current pregnancy and any previous contact) | | | |  | |  |
| **Social Services involvement** | Circumstances of referral (if known)   - Referrer - Time of referral - Main reason for referral - Length of time between referral and actual involvement from Social Services - Any referrals rejected | | |  | |  |
|  | Involvement for: 1) Mother; 2) Baby; 3) Both | | |  | |  |
|  | Involvement prior to death (Yes/No) | | |  | |  |
|  | Highest level of involvement: (CIN/CP/PLO/ICO/PPO/s20/other) | | |  | |  |
|  | Child taken into care  (Yes/No) – if yes, please give details if this was prior or after maternal death | | |  | |  |
|  | Previous involvement, incl. removals (Yes/No) – if yes, please give details | | |  | |  |
| **Thematic Guidance for Case Note Review with prompts***** | | | | | | |
| **1. Women should have access to all relevant services as early as possible, with clear communication between agencies**  **(BC Charter Principle 1, 4 & 6)** | | | | | | |
| - Was there a confident and skilled conversation about potential safeguarding concerns at booking? - Was there a prompt referral to LA as soon as safeguarding concerns were identified? - Was there evidence or clear documentation of thresholds when referrals were sent? - Was there a timely offer of specialist support (1st trim)? - Were professionals’ concerns and plans shared with parents, while continually checking parents’ understanding? - Was there consistent and frequent multi-agency communication? - Were services joined-up, co-located where possible? - Did the woman have access to all relevant universal and specialist services required? - Was the woman offered or signposted to antenatal education classes? - Was the woman excluded from any services/care on the grounds of social services involvement? If so, which services? - Was there any evidence of MDT safety planning prior to discharge for women subject to Domestic Abuse? | | | | | | |
| **2. Women should receive support that is specialist and continuous during pregnancy, birth and early motherhood**  **(BC Charter principles 1)** | | | | | | |
| - Was there involvement of staff with expertise in safeguarding? - Were there any perinatal mental health practitioners involved? - Was the woman seen in a specialist pathway for women with social services involvement? - Was there evidence of flexibility in appointments? - Was there evidence of professional understanding of risk, for instance re coercive control. - Was the woman prioritised for Continuity of Carer? - If fragmented care, give summary or examples - In case of domestic abuse, did she have access to appropriate support, such as an IDVA? - In case of substance misuse, did she have access to specialist addiction services? - Was there an assertive outreach approach taken to the woman’s postnatal care? - Was the postnatal offer extended beyond 10days PN? - Was information shared clearly with GP, health visitors and other services involved? | | | | | | |
| **3. Women should receive support that is women-centred, holistic and culturally appropriate (BC Charter principle 2 & 9)** | | | | | | |
| - Was there any consideration given to the woman’s specific learning and communication needs or preferences (incl. interpreter requirements)? - Was there evidence of parental distrust of services? If so, what actions did maternity staff undertake to mitigate this? - Was care oriented towards the baby and not towards the mother? - Did the woman have opportunities to discuss issues without partner present? - Was the mother’s privacy, dignity and confidentiality respected? - Was supervision proportionate to the level of risk and concern? - Was the mother supported to attend the court hearing, especially if still recovering in hospital? | | | | | | |
| **4. Women should receive support that is trauma-informed and trauma-responsive (BC Charter principles 3)** | | | | | | |
| - Is there evidence that the impact of previous social services involvement or previous removals was considered? - Was the mother’s history of trauma taken into account? - Was challenging or avoidant behaviour viewed through a lens of possible past trauma? - Did the woman experience any form of racism? - Did the woman experience any discrimination related to protected characteristics? - Were efforts made to minimise or mitigate the risk of re-triggering trauma? - In case of domestic abuse, was there evidence of victim-blaming (i.e. women being held accountable for partner’s behaviour?) - Was sensitivity shown regarding the information shared with other professionals/in records and its potential impact on parents? | | | | | | |
| **5. Women should receive support that is responsive to their specific needs, including mental health support? (BC Charter principles 5, 7, 9 and 10)** | | | | | | |
| - Were referrals due to MH concerns made at the earliest opportunity? - Was the woman supported by MH professionals to understand any MH diagnoses? - Did she have access to appropriate MH provisions? - Were transitions between MH services based on need? - Was there a protected postpartum period immediately after birth to allow for recovery and bonding? - If baby was on NICU, was access to baby equivalent to other mothers without SSI? - Were the immediate basic and emotional needs of the mother prior to hospital discharge checked? - Was community support put in place prior to hospital discharge? - Was there access to services and professional support post-removal? - If no longer eligible post-removal, was there timely alternative support made available? - Was the woman prioritised to ensure timely offer of MH support post separation? | | | | | | |
| **6. Women should be having their birth preferences and parenting choices respected (BC Charter Principles 8 and 10)** | | | | | | |
| - Were LA plans re birth arrangements and plan for baby shared by 30w gestation? - Were parents involved in producing the birth arrangements, incl. choice of birthing partner? - Were women’s preferences around birth and early PN period discussed and documented? - Were women’s preferences around birth and early PN period followed? - Was she supported by a birth partner? - Was the mother given maximum opportunities to parent her baby, in the way she preferred (e.g. feeding choices, skin-to-skin)? - Was there any consideration given that the woman needed space and time to prepare emotionally and practically for a possible separation? - Was there any consideration for continuing post-separation bonding (eg. creating mementos etc)?   Was mother given clear information about future contact arrangements prior to separation? | | | | | | |
| **7. Women should have their rights upheld through clear ways to express concerns, challenge inaccuracies and make complaints about unfair or poor practice (BC Charter Principle 14)** | | | | | | |
| - Did the woman challenge any information collected or presented due to inaccuracy? - Was there any information shared re complaints procedures? - Was she supported by maternity staff to raise concerns with other members of the multi-professional team around her?   Did the woman have access to an advocate or some independent support | | | | | | |
| **8. Additional: Was there any evidence of judgement or bias in care or support received?** | | | | | | |
| Please provide any examples of language indicative of judgement or bias related to social services involvement or complex social factors | | | | | | |

* Cosstick et al., 2022: doi: 10.1016/j.eclinm.2022.101587; Birth Companions 2019 report

** Yes: asked and present / No: asked but not present / NA: not asked or no information available

*** Guidance taken from Birth Companions Charter for women with involvement from children’s social care (2023) and Born Into Care best practice guidance for when the state intervenes at birth (2023)
